# Leveraging Single-Cell Multi-Omics to Decode Tumor Microenvironment Diversity and Therapeutic Resistance

**DOI:** 10.3390/ph18010075

**Published:** 2025-01-10

**Authors:** Hussein Sabit, Borros Arneth, Timothy M. Pawlik, Shaimaa Abdel-Ghany, Aysha Ghazy, Rawan M. Abdelazeem, Amany Alqosaibi, Ibtesam S. Al-Dhuayan, Jawaher Almulhim, Noof A. Alrabiah, Ahmed Hashash

**Affiliations:** 1 Department of Medical Biotechnology, College of Biotechnology, Misr University for Science and Technology, P.O. Box 77, Giza 3237101, Egypt; 2 Institute of Laboratory Medicine and Pathobiochemistry, Molecular Diagnostics, Hospital of the Universities of Giessen and Marburg (UKGM), Philipps University Marburg, Baldingerstr. 1, 35043 Marburg, Germany; 3 Department of Surgery, The Ohio State University, Wexner Medical Center, Columbus, OH 43210, USA; 4 Department of Environmental Biotechnology, College of Biotechnology, Misr University for Science and Technology, P.O. Box 77, Giza 3237101, Egypt; 5 Department of Agricultural Biotechnology, College of Biotechnology, Misr University for Science and Technology, P.O. Box 77, Giza 3237101, Egypt; 6 Department of Biology, College of Science, Imam Abdulrahman bin Faisal University, P.O. Box 1982, Dammam 31441, Saudi Arabia; 7 Department of Biological Sciences, King Faisal University, Alahsa 31982, Saudi Arabia; 8 Department of Biomedicine, Texas A&M University, College Station, TX 77843, USA

**Keywords:** single-cell multi-omics, tumor microenvironment (TME), cancer therapeutic resistance, immune evasion, metabolic reprogramming, personalized cancer therapy

## Abstract

Recent developments in single-cell multi-omics technologies have provided the ability to identify diverse cell types and decipher key components of the tumor microenvironment (TME), leading to important advancements toward a much deeper understanding of how tumor microenvironment heterogeneity contributes to cancer progression and therapeutic resistance. These technologies are able to integrate data from molecular genomic, transcriptomic, proteomics, and metabolomics studies of cells at a single-cell resolution scale that give rise to the full cellular and molecular complexity in the TME. Understanding the complex and sometimes reciprocal relationships among cancer cells, CAFs, immune cells, and ECs has led to novel insights into their immense heterogeneity in functions, which can have important consequences on tumor behavior. In-depth studies have uncovered immune evasion mechanisms, including the exhaustion of T cells and metabolic reprogramming in response to hypoxia from cancer cells. Single-cell multi-omics also revealed resistance mechanisms, such as stromal cell-secreted factors and physical barriers in the extracellular matrix. Future studies examining specific metabolic pathways and targeting approaches to reduce the heterogeneity in the TME will likely lead to better outcomes with immunotherapies, drug delivery, etc., for cancer treatments. Future studies will incorporate multi-omics data, spatial relationships in tumor micro-environments, and their translation into personalized cancer therapies. This review emphasizes how single-cell multi-omics can provide insights into the cellular and molecular heterogeneity of the TME, revealing immune evasion mechanisms, metabolic reprogramming, and stromal cell influences. These insights aim to guide the development of personalized and targeted cancer therapies, highlighting the role of TME diversity in shaping tumor behavior and treatment outcomes.

## 1. Introduction

Cancer represents a disease marked by uncontrolled cell growth, in which these transformed cells evolve under natural selection pressures [1]. The progression of cancer and resistance to treatment are strongly influenced by the tumor microenvironment (TME), a dynamic and intricate network comprising cancer cells, stromal cells, immune cells, and the extracellular matrix (ECM), all working together to support tumor development and metastasis [2]. The complexity and diversity of the TME represent unique challenges and opportunities to treat cancer. Breakthroughs in single-cell multi-omics technologies have allowed researchers to gain unprecedented insights into the TME, demonstrating its critical role in cancer growth and resistance to treatments.

A major component of the TME is the cancer-associated fibroblasts (CAFs). These fibroblasts are key players in remodeling the ECM, secreting growth factors, and modulating the immune system, significantly affecting tumor expansion, spread, and treatment response [3]. For example, CAFs release cytokines such as TGF-β, which drives the epithelial–mesenchymal transition (EMT) in cancer cells, thereby boosting their ability to migrate and invade [4].

Immune cells within the TME, such as T cells, macrophages, and neutrophils, also have critical roles. Tumor-associated macrophages (TAMs) can foster tumor growth by encouraging blood vessel formation, tissue remodeling, and immune suppression. On the other hand, cytotoxic T lymphocytes (CTLs) and natural killer (NK) cells can attack and destroy cancer cells, although their activity is often dampened in the TME due to various immune evasion mechanisms deployed by tumors [5,6].

The introduction of single-cell multi-omics has dramatically enhanced our understanding of the TME by allowing the simultaneous examination of several molecular layers at a single-cell level. Methods like single-cell RNA sequencing (scRNA-seq), single-cell ATAC-seq (Assay for Transposase-Accessible Chromatin), and single-cell proteomics have exposed the TME’s vast heterogeneity [7]. These techniques deliver detailed maps of gene expression, chromatin accessibility, and protein levels in individual cells, offering new perspectives on the cellular interactions and states that propel cancer growth and resistance.

For instance, scRNA-seq has uncovered various immune cell types within tumors, including exhausted T cells marked by an elevated expression of inhibitory receptors like PD-1 and CTLA-4 [8]. These discoveries have major implications for immunotherapy, as they identify potential targets for immune checkpoint inhibitors. Additionally, single-cell analyses have pinpointed distinct metabolic profiles in both cancer and stromal cells, illuminating how altered metabolism fuels tumor growth in low-oxygen conditions [9].

The knowledge obtained from single-cell multi-omics studies of the TME is set to revolutionize cancer treatment. Mapping tumor and microenvironment diversity enables the design of more targeted and effective therapies. For instance, focusing on specific CAF subsets or their secretions could boost the effectiveness of existing treatments by bypassing resistance mechanisms [10]. Likewise, modifying the immune landscape within the TME can enhance immunotherapies by reactivating exhausted T cells and mitigating immunosuppression [11].

Moreover, single-cell multi-omics provides a foundation for personalized medicine by identifying specific molecular changes driving cancer in each patient. This precision approach allows for the selection of therapies that are more likely to succeed, reducing resistance risks and improving outcomes [6]. Understanding the metabolic interactions in the TME also opens doors to metabolic targeting, which can cut off energy supplies to cancer cells and amplify the impact of chemotherapy and radiotherapy [12].

Future research should prioritize integrating single-cell multi-omics data to achieve a complete understanding of the TME. Long-term studies tracking the TME’s changes over time and in response to treatment could uncover early resistance biomarkers and guide adaptive therapy strategies [13]. Spatial transcriptomics, which merges single-cell sequencing with spatial context, can chart the cellular layout within the TME, demonstrating how physical location affects tumor behavior [14].

Ultimately, single-cell multi-omics technologies have unlocked new opportunities in cancer research, offering deep insights into the TME’s influence on cancer progression and resistance. Leveraging these insights allows researchers to create innovative, personalized treatment strategies, leading to better outcomes for patients. As these technologies advance, their potential to transform cancer therapy continues to grow, marking the dawn of a new era in oncology.

This review highlights the potential of single-cell multi-omics to uncover the cellular and molecular heterogeneity within the tumor microenvironment (TME). By elucidating mechanisms such as immune evasion, metabolic reprogramming, and the influence of stromal cells, these approaches provide a deeper understanding of TME complexity. Such insights are instrumental in shaping the development of personalized and targeted cancer therapies, emphasizing the critical role of TME diversity in determining tumor behavior and influencing therapeutic outcomes.

## 2. Tumor Microenvironment (TME)

**Figure 1:**
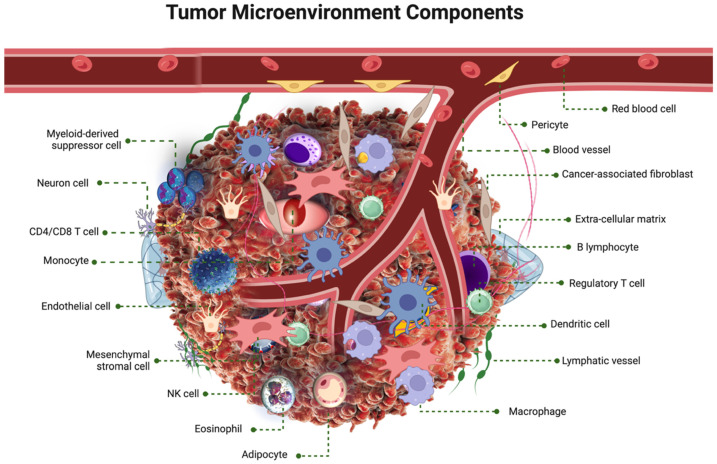
The TME components and their interactions. The TME comprises CAFs, which modify the ECM and secrete growth factors; immune-suppressive cells like myeloid-derived suppressor cells (MDSCs) and regulatory T cells (Tregs); and anti-tumor immune cells such as CD4/CD8 T cells and NK cells, whose activity is often hindered. Additionally, endothelial cells, pericytes, and blood vessels support angiogenesis and metastasis, while lymphatic vessels assist in metastasis and immune regulation. Adipocytes and mesenchymal stromal cells contribute metabolic support, promoting tumor growth, while neurons emphasize the growing role of neuro-immune interactions. This complex network demonstrates how the TME shapes tumor behavior and resistance to therapy.

The tumor microenvironment (TME) is a highly dynamic and complex ecosystem surrounding tumor cells, playing an essential role in cancer growth and progression (Figure 1). The TME includes various cellular and non-cellular elements that constantly interact with cancer cells, impacting their behavior and response to treatments [2].

At the core of the tumor microenvironment (TME) are cancer cells, which are characterized by unchecked growth and an ability to bypass normal regulatory mechanisms. These cells undergo genetic and epigenetic changes that drive their proliferation, survival, and metastatic potential [15]. Surrounding these cancer cells are stromal cells, including fibroblasts, endothelial cells, and pericytes, which provide structural support within the TME. Cancer-associated fibroblasts (CAFs) play a crucial role by secreting growth factors, cytokines, and extracellular matrix (ECM) components that create a favorable environment for tumor growth. Endothelial cells further contribute to this environment by promoting angiogenesis, which is essential for tumor growth and metastasis [3].

The TME also hosts a diverse array of immune cells such as macrophages, lymphocytes, dendritic cells, and neutrophils. Tumor-associated macrophages (TAMs) can have pro-tumorigenic effects, such as promoting angiogenesis, tissue remodeling, and suppressing the immune response [5]. On the other hand, cytotoxic T lymphocytes (CTLs) and natural killer cells, which are capable of destroying cancer cells, are often rendered ineffective within the TME [16]. The ECM, composed of proteins like collagen, elastin, and fibronectin, provides a structural scaffold for tissues. In the TME, the ECM undergoes significant remodeling through the action of matrix metalloproteinases (MMPs), which are produced by both cancer and stromal cells, facilitating cancer cell invasion and metastasis [17].

### 2.1. The Role of TME in Cancer Biology

The TME significantly influences cancer biology, affecting everything from tumor initiation to metastasis. Understanding the interactions between cancer cells and the various TME components is vital for developing effective treatments.

**Figure 2:**
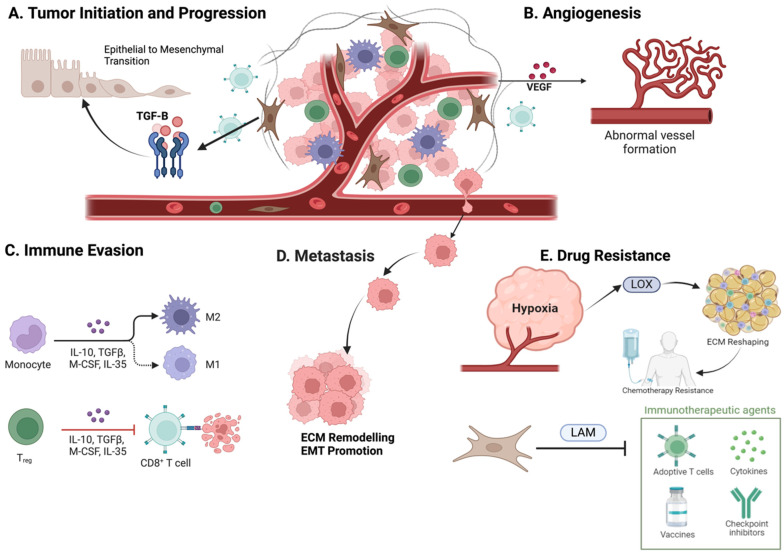
Key processes in tumor progression and therapeutic resistance. The illustration highlights five critical processes within the TME that drive tumor progression and resistance to therapy: (**A**) EMT-driven invasiveness, (**B**) VEGF-promoted angiogenesis, (**C**) immune evasion through immune cell manipulation, (**D**) ECM restructuring leading to metastasis, and (**E**) the development of resistance due to hypoxia and ECM changes.

The interactions between cancer cells and the tumor microenvironment (TME) are critical in driving tumor initiation, growth, and progression (Figure 2). Cancer-associated fibroblasts (CAFs) within the TME secrete growth factors like TGF-β, which induce an epithelial–mesenchymal transition (EMT), enhancing the migratory and invasive potential of cancer cells [4]. Chronic inflammation, often fueled by immune cells within the TME, can further promote genetic mutations and tumor growth [18]. For tumors to grow beyond a certain size, angiogenesis is essential, and the TME drives this process through the release of pro-angiogenic factors such as VEGF from cancer cells, CAFs, and tumor-associated macrophages (TAMs) [19]. This newly formed vasculature not only nourishes the tumor but also provides pathways for metastasis. Additionally, the TME plays a central role in immune evasion by creating an immunosuppressive environment that suppresses the activity of cytotoxic T lymphocytes (CTLs) and natural killer (NK) cells. TAMs, for instance, secrete IL-10 and TGF-β, which inhibit anti-tumor immune responses [20], and the expression of immune checkpoint proteins like PD-L1 on cancer cells further dampens T cell function [11].

The TME also facilitates metastasis through mechanisms such as ECM remodeling and EMT, allowing cancer cells to detach from the primary tumor, invade nearby tissues, and establish secondary tumors in distant locations [21]. Moreover, the TME contributes to therapeutic resistance, which is a major challenge in cancer treatment, as hypoxia within the TME can trigger the expression of drug-resistance genes, and stromal cells may secrete factors that protect cancer cells from chemotherapy and radiation therapy [22].

Studies have shown that interactions between tumor-associated macrophages and cancer-associated fibroblasts (CAFs) can enable cancer cells to develop resistance to gemcitabine and paclitaxel in pancreatic and breast cancers, primarily through IGF-1/2 signaling pathways [23,24]. Additionally, while natural killer (NK) cells are highly effective in eliminating cancer cells, their cytotoxic activity is significantly diminished by exposure to the TGF-β secreted by CAFs. This occurs through the miR-183-mediated disruption of DAP12 transcription, ultimately enhancing cancer cell survival and resistance to chemotherapy [25].

### 2.2. Cellular Heterogeneity in the TME

**Figure 3:**
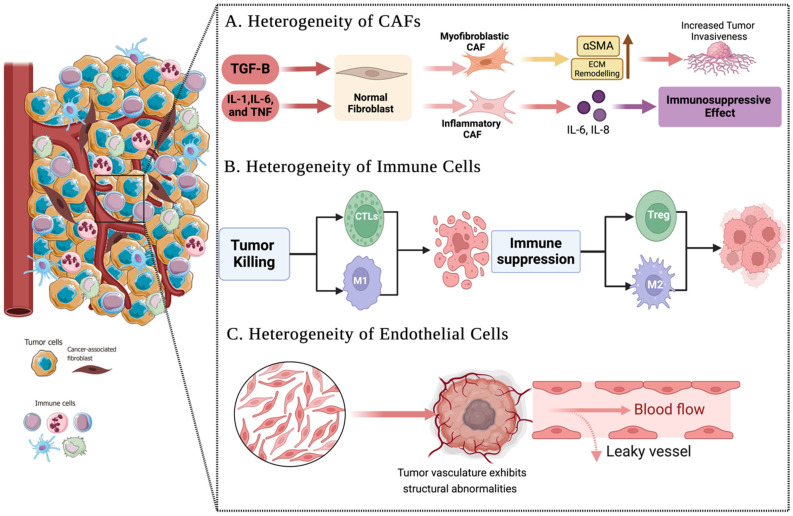
Heterogeneity in the TME and cancer-associated fibroblasts (CAFs). The figure showcases the diverse cell types in the TME, including myofibroblastic CAFs (myCAFs) and inflammatory CAFs (iCAFs) with distinct roles; the dual nature of immune cells like CTLs and Tregs; and the role of abnormal endothelial cells in fostering an environment conducive to tumor growth.

The TME is highly heterogeneous, comprising a wide variety of cell types, including cancer-associated fibroblasts (CAFs), immune cells, and endothelial cells. This cellular diversity profoundly influences tumor behavior and treatment outcomes (Figure 3).

CAFs can originate from multiple sources, including resident fibroblasts, mesenchymal stem cells, or epithelial cells undergoing EMT under tumor influence [3]. CAFs can be categorized into subpopulations based on their functions and phenotypes. Myofibroblastic CAFs (myCAFs) are characterized by high α-SMA expression and are key players in ECM remodeling, which enhances tumor invasiveness [26]. On the other hand, inflammatory CAFs (iCAFs) secrete inflammatory cytokines like IL-6 and IL-8, fostering an immunosuppressive environment that aids tumor progression [27].

The functional heterogeneity of CAFs is regulated by specific signaling pathways and their cellular origins. For example, IL-1-induced JAK/STAT signaling promotes iCAF formation, while TGF-β signaling opposes this process, encouraging the differentiation of myCAFs [27]. This interplay underscores the complexity of CAF functions and their potential as therapeutic targets.

The immune cells within the TME also display significant heterogeneity. CTLs and Tregs represent two T cell subtypes with opposing roles. CTLs are vital for anti-tumor immunity but are often suppressed within the TME [16]. In contrast, Tregs suppress immune responses and facilitate tumor immune evasion, correlating with worse prognoses [28]. Similarly, TAMs exhibit functional plasticity, with M1-like TAMs promoting anti-tumor activity, while M2-like TAMs enhance tumor growth by driving angiogenesis, tissue remodeling, and immune suppression [29].

### 2.3. Single-Cell Analysis of Immune Cell Populations in the TME

Single-cell multi-omics technologies have transformed our understanding of biological systems by providing high-resolution views of cellular diversity and functionality. By integrating genomic, transcriptomic, proteomic, and metabolomic data at the single-cell level, this approach is particularly valuable in studying the TME. It offers detailed insights into the molecular mechanisms of cancer, uncovering rare cell populations and crucial interactions driving therapy resistance and disease progression. This advancement not only deepens our knowledge of tumor biology but also supports the development of personalized treatment strategies tailored to each patient’s unique tumor landscape [30,31].

Single-cell RNA sequencing (scRNA-seq) has especially revolutionized the profiling of immune cells within the TME, allowing for the dissection of immune cell heterogeneity and the identification of rare and functionally distinct subpopulations that were previously undetectable [32]. The throughput of single-cell RNA sequencing (scRNA-seq) has significantly improved, allowing the analysis of hundreds of thousands of cells per experiment while reducing costs. Consequently, the adoption of scRNA-seq in research has grown, with advanced methods like microfluidic-, microwell-, and droplet-based technologies, in situ barcoding, and spatial transcriptomics driving this trend [33]. The main steps of scRNA-seq include single-cell isolation, cell lysis, reverse transcription (RNA to cDNA conversion), cDNA amplification, and library preparation. Among these, single-cell capture, reverse transcription, and cDNA amplification are the most technically demanding. Advances in sequencing platforms have also led to rapid and diverse improvements in RNA-seq library preparation techniques [34,35].

For example, scRNA-seq has revealed diverse T cell populations within the TME, including exhausted, cytotoxic, and regulatory T cells (Tregs). These findings highlight how the immune landscape within tumors is not homogeneous but rather a mosaic of cell types with varying states of activation and function [36].

Another study used single-cell RNA sequencing (scRNA-seq) to map the immune landscape of hepatocellular carcinoma (HCC) across tumors, lymph nodes, blood, and ascites in 16 patients. Tumors were enriched with immunosuppressive regulatory T cells (Tregs) and exhausted CD8+ T cells (Tex), while proliferative T cells were abundant in ascites. Two macrophage states were identified: MDSC-like macrophages and TAM-like macrophages, the latter being linked to poor prognosis and inflammatory markers like SLC40A1 and GPNMB. Migratory LAMP3+ dendritic cells (DCs) were also identified, interacting with Tregs and Tex through immune checkpoints like PD-L1 and promoting T cell dysfunction. These findings reveal mechanisms of immune suppression, migration, and evasion, offering therapeutic insights [37].

Beyond T cells, scRNA-seq has provided insights into the complexity of myeloid cells in the TME, such as macrophages and dendritic cells. These cells can either promote tumor growth through immunosuppressive actions or boost anti-tumor immunity. For instance, TAMs exhibit a spectrum of phenotypic states ranging from pro-inflammatory M1 macrophages to immunosuppressive M2 macrophages, highlighting the delicate balance between tumor-promoting and tumor-suppressing functions within the TME [38].

For instance, using single-cell RNA sequencing (scRNA-seq) in a study, distinct populations of T cells, B cells, and myeloid cells were identified, each contributing uniquely to tumor progression and immune modulation [39]. Exhausted CD8+ T cells (subclusters C8 and C9) were marked by inhibitory receptors like PDCD1 (PD-1) and LAG3, significantly impairing their cytotoxic activity within tumors. Regulatory CD4+ T cells (Tregs) also contributed to immune suppression through markers like FOXP3 and CTLA4. While naïve T cells were prevalent in normal tissues, tumor-associated regulatory B cells (Bregs) promoted progression by suppressing T cell function and enhancing angiogenesis pathways [40].

Myeloid cells exhibited notable heterogeneity, with tumor-associated macrophages (TAMs) adopting M2-like pro-tumoral phenotypes, enriched in angiogenesis and glycolysis pathways. TAMs in subcluster C2 were strongly associated with tumor progression, while dendritic cells (DCs) in subcluster DC2 were crucial for antigen presentation and T cell activation [41]. Monocytes, primarily found in the blood, infiltrated tissues and differentiated into macrophages. This complex interplay among immune subtypes underscores the immunosuppressive nature of the TME and provides valuable insights into potential therapeutic strategies, such as targeting exhausted T cells or modulating TAM and Breg activity to enhance anti-tumor immunity [42].

**Figure 4:**
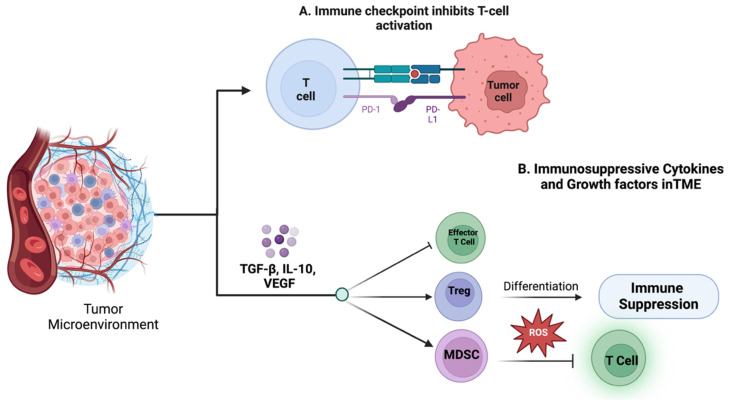
Immune evasion mechanisms in the tumor microenvironment (TME). (**A**) Immune checkpoint inhibition: Tumor cells exploit immune checkpoints, such as PD-1/PD-L1 interactions, to suppress T cell activation and evade immune responses. This pathway directly impairs effector T cell function, allowing tumors to grow unchecked. (**B**) Immunosuppressive cytokines and growth factors in TME: Tumor-secreted factors, including TGF-β, IL-10, and VEGF, induce differentiation of immune-suppressive cell populations such as regulatory T cells (Tregs) and myeloid-derived suppressor cells (MDSCs). These cells release reactive oxygen species (ROS) and other suppressive signals, further promoting immune suppression and hindering T cell-mediated tumor elimination.

A key hallmark of cancer is its ability to evade immune surveillance. Several mechanisms enable this immune evasion, contributing to the immunosuppressive TME (Figure 4). One significant mechanism involves the expression of immune checkpoint molecules like PD-1, CTLA-4, and PD-L1, which inhibit T cell activation, allowing cancer cells to escape immune detection. Recent single-cell studies have mapped the dynamics of these checkpoints within the TME, uncovering their role in sustaining immune suppression [43].

Another study utilized single-cell RNA sequencing (scRNA-seq) to define two major tumor microenvironment (TME) patterns in lung adenocarcinoma, validated for prognostic relevance using bulk RNA-seq data from 533 patients in The Cancer Genome Atlas (TCGA) cohort. The N^3^MC (normal-like microenvironment) pattern, enriched in normal-like myofibroblasts and non-inflammatory immune cells, was associated with better overall survival (hazard ratio [HR]: 0.50; 95% confidence interval [CI]: 0.35–0.71, *p* = 0.0001). Conversely, the CP^2^E (cancer-progressive environment) pattern, characterized by cancer-associated myofibroblasts, pro-inflammatory macrophages, and exhausted T cells, correlated with reduced survival (HR: 2.0; CI: 1.4–2.9, *p* < 0.001). These patterns were derived from scRNA-seq data comprising 114,489 high-quality single-cell transcriptomes, linking cellular diversity to patient outcomes [44].

The Single-cell Assay for Transposase-Accessible Chromatin using sequencing (scATAC-seq) is a powerful tool for exploring chromatin accessibility at the single-cell level, enabling the identification of regulatory DNA elements and transcription factor activity. In cancer research, it has been instrumental in dissecting tumor heterogeneity, revealing regulatory networks in malignant cells, and understanding the immune microenvironment. Specifically, in the tumor microenvironment (TME), scATAC-seq provides valuable insights into the epigenetic landscapes of immune cells, such as exhausted T cells, which play a critical role in tumor progression and responses to immunotherapy. By offering an unbiased view of chromatin dynamics, this method facilitates the discovery of key regulatory elements across diverse cell populations [45].

A study used scATAC-seq to analyze over 200,000 single cells from blood and basal cell carcinoma (BCC), including 37,818 tumor biopsy cells from pre- and post-PD-1 blockade therapy. Distinct immune, stromal, and tumor cell clusters were identified, with a notable post-therapy expansion of CD8+ exhausted T cells (TEx), constituting over 90% of the TEx population. Chromatin remodeling linked to therapy response was evident, with 4598 TEx-specific cis-elements identified. Additionally, a shared regulatory program between TEx and T follicular helper (Tfh) cells was observed, driven by transcription factors BATF, IRF4, and NFATC1. These findings highlight scATAC-seq’s role in uncovering regulatory mechanisms in the tumor microenvironment and advancing cancer immunotherapy [46].

Moreover, the TME is rich in immunosuppressive cytokines and growth factors, such as TGF-β, IL-10, and VEGF, which further enhance immune evasion. These factors inhibit the activity of effector T cells while promoting the development of Tregs and myeloid-derived suppressor cells (MDSCs). For example, IL-10 and TGF-β encourage Treg differentiation, which in turn suppresses anti-tumor immune responses [47]. Similarly, MDSCs, which are prevalent in the TME, can impair T cell function by producing reactive oxygen species (ROS) and nitric oxide (NO), further weakening the immune response [48].

The tumor microenvironment (TME) enables immune evasion through hypoxia, metabolic competition, and structural barriers. Hypoxia-driven HIF-1α upregulates immune checkpoints like PD-L1, impairs CTLs and NK cells, and recruits regulatory T cells. Cancer cells produce lactate, suppressing immune cells and attracting MDSCs. The disorganized vasculature limits immune infiltration, while extracellular vesicles deliver suppressive molecules. Additionally, cancer cells outcompete immune cells for essential nutrients, weakening their function. These mechanisms create an immunosuppressive niche that promotes tumor growth and therapy resistance, highlighting the need for targeted strategies [40].

Insights gained from the single-cell analysis of the TME’s immune landscape have opened new avenues for targeted immunotherapies. One promising approach involves immune checkpoint inhibitors (ICIs) that block PD-1, PD-L1, and CTLA-4, reactivating exhausted T cells and enhancing anti-tumor immunity. ICIs have demonstrated significant efficacy to treat cancers like melanoma and non-small cell lung cancer [49]. Additionally, targeting the immunosuppressive cytokine network is another emerging strategy. Inhibitors targeting TGF-β and IL-10 are currently in clinical trials, aiming to reduce immunosuppression and boost T cell activity [11]. Moreover, targeting specific immunosuppressive cell populations, such as MDSCs and Tregs, represents a novel therapeutic approach, with drugs and antibodies designed to deplete or modulate these cells under investigation [50].

Further innovations include chimeric antigen receptor T cell (CAR-T) therapies and cancer vaccines that harness the patient’s immune system to target specific cancer antigens. These therapies are increasingly being refined based on molecular and cellular profiles obtained from single-cell analyses, enhancing their efficacy while minimizing side effects [51]. Ongoing research in single-cell multi-omics holds great promise to develop more personalized and effective cancer treatments, ultimately improving patient outcomes.

### 2.4. Metabolic Reprogramming in the TME

Metabolic reprogramming within the TME is another critical factor influencing tumor progression and therapeutic resistance. The metabolic interactions between cancer cells and stromal cells are key to supporting tumor growth and impact the efficacy of therapies.

Cancer cells undergo metabolic shifts to sustain rapid proliferation and survival under stressful conditions, a phenomenon known as metabolic reprogramming. This process includes an increased reliance on glycolysis, glutaminolysis, and fatty acid synthesis [12]. However, cancer cells do not operate in isolation—they engage in metabolic crosstalk with stromal cells, like CAFs, immune cells, and endothelial cells, creating a cooperative environment that supports tumor growth.

A notable metabolic interaction within the TME is the “reverse Warburg effect”. In contrast to the classical Warburg effect—where cancer cells depend heavily on glycolysis even when oxygen is available—the reverse Warburg effect involves stromal cells, particularly CAFs, undergoing aerobic glycolysis to produce lactate and pyruvate. Cancer cells then absorb these metabolites and use them for oxidative phosphorylation (OXPHOS), enabling sustained ATP production and tumor growth under challenging conditions [52].

Additionally, adipocytes in the TME supply fatty acids to cancer cells through lipolysis. These fatty acids are then used in beta-oxidation, which is essential for energy production and supports the rapid division of cancer cells [53]. This metabolic symbiosis allows cancer cells to adapt to nutrient fluctuations within the TME.

Metabolic reprogramming also plays a pivotal role in therapeutic resistance. For example, the acidic environment created by enhanced glycolysis in cancer cells can decrease the uptake and effectiveness of weakly basic chemotherapeutic drugs [54]. Furthermore, altered metabolic states within the TME can affect immune cell functionality, further contributing to resistance. High lactate levels, a byproduct of aerobic glycolysis, can inhibit CTLs and NK cells, compromising anti-tumor immunity [6].

Cancer cells can also develop resistance to targeted therapies through metabolic adaptations. For example, resistance to BRAF inhibitors in melanoma has been linked to a shift towards increased mitochondrial biogenesis and reliance on OXPHOS, allowing cancer cells to survive despite inhibition of the MAPK pathway [55]. Similarly, breast cancer cells rely more on fatty acid synthesis and storage, contributing to resistance against HER2-targeted therapies [56].

A crucial aspect of metabolic reprogramming is its role in maintaining cancer stem cells (CSCs), which are a subpopulation of cells with self-renewal capabilities that drive tumor recurrence [57]. CSCs often exhibit a unique metabolic profile, relying heavily on OXPHOS and fatty acid oxidation, which makes them resistant to conventional therapies that target rapidly dividing cells. Targeting the metabolic vulnerabilities of CSCs is an emerging strategy for overcoming resistance and preventing tumor relapse [58].

Understanding these metabolic interactions and their contribution to therapeutic resistance is key to developing more effective cancer treatments. Future research should focus on identifying the specific metabolic pathways involved and designing targeted therapies that disrupt these networks, ultimately leading to better patient outcomes.

### 2.5. Therapeutic Resistance Mediated by the TME

**Figure 5:**
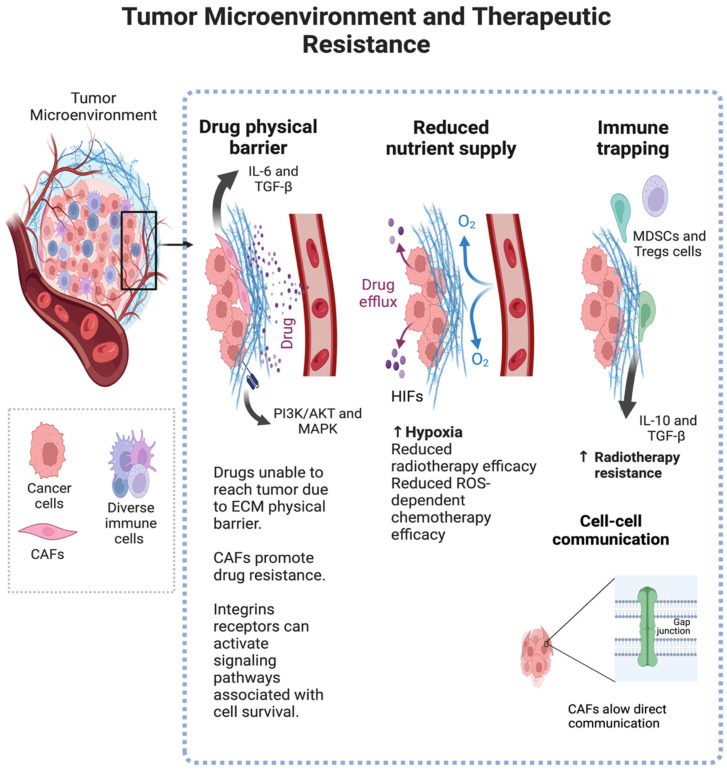
The tumor microenvironment (TME) and therapeutic resistance. This figure highlights the complex ways the tumor microenvironment (TME) contributes to therapeutic resistance. The TME, made up of cancer-associated fibroblasts (CAFs), immune cells, and the extracellular matrix (ECM), poses significant barriers to treatment. CAFs alter the ECM using cytokines like IL-6 and TGF-β, creating a physical barrier that hampers drug delivery while triggering survival pathways through integrin receptors. In nutrient-deprived areas, hypoxia-induced factors (HIFs) weaken the effectiveness of radiotherapy and chemotherapy by promoting drug efflux and resistance. The TME also recruits immune-suppressive cells like myeloid-derived suppressor cells (MDSCs) and regulatory T cells (Tregs), which release IL-10 and TGF-β, further stalling the immune response and increasing resistance to treatment. Additionally, CAFs communicate directly with tumor cells through gap junctions, helping the tumor thrive. These interlinked mechanisms within the TME highlight the complexity of cancer resistance, presenting crucial targets for overcoming therapeutic challenges.

The tumor microenvironment (TME) plays a critical role in the development of therapeutic resistance in cancer (Figure 5). The interactions among various components within the TME significantly influence how tumors respond to treatments, including chemotherapy, radiotherapy, and targeted therapies.

Chemotherapy resistance: The TME contributes to chemotherapy resistance through several mechanisms. One major factor is the presence of hypoxia within tumors. Under low oxygen conditions, hypoxia-inducible factors (HIFs) are stabilized, leading to the activation of genes that promote angiogenesis, glycolysis, and resistance to cell death (apoptosis) [59]. This hypoxic environment can reduce the efficacy of chemotherapy drugs that rely on oxygen to generate reactive oxygen species (ROS) for DNA damage [60]. Additionally, CAFs in the TME secrete growth factors and cytokines like IL-6 and TGF-β, which activate survival pathways in cancer cells, further contributing to drug resistance [61]. The dense ECM produced by CAFs also acts as a physical barrier, preventing chemotherapeutic agents from penetrating deep into the tumor [62].

Radiotherapy resistance: Hypoxia within the TME also contributes to radiotherapy resistance, as oxygen is a key radiosensitizer that enhances the DNA-damaging effects of radiation. In hypoxic conditions, HIFs drive the expression of genes that promote DNA repair and reduce oxidative stress, making cancer cells less susceptible to radiation [63]. Furthermore, the immunosuppressive environment within the TME, driven by cells like MDSCs and Tregs, can inhibit the activation of anti-tumor immune responses following radiotherapy, reducing its effectiveness [64]. Additionally, cytokines such as IL-10 and TGF-β secreted by TME components further dampen the immune response, contributing to radioresistance [65].

Targeted therapy resistance: The TME can also undermine the effectiveness of targeted therapies. For instance, stromal cells may secrete growth factors and cytokines that activate alternative survival pathways in cancer cells, allowing them to bypass the inhibitory effects of targeted therapies. An example is the secretion of hepatocyte growth factor (HGF) by stromal cells, which can activate the MET signaling pathway, providing a survival route for cancer cells even when EGFR-targeted therapies are applied [66]. Additionally, the ECM can sequester and inactivate targeted drugs, reducing their availability to cancer cells. Hyaluronan, a component of the ECM, can bind to therapeutic antibodies, decreasing their efficacy [67]. The stiffness and density of the ECM also influence how well targeted therapies can penetrate the tumor, contributing to resistance [68].

### 2.6. Role of ECM and Cell–Cell Communication in Resistance

The extracellular matrix (ECM) plays a pivotal role in therapeutic resistance by providing structural support and delivering biochemical signals to cancer cells. The ECM consists of proteins, glycoproteins, and proteoglycans that create a scaffold for tissue architecture. In the TME, CAFs actively remodel the ECM, altering its composition and mechanical properties [68].

The ECM contributes to therapeutic resistance through several mechanisms. First, its dense and fibrous nature can impede the penetration of therapeutic agents, limiting their access to cancer cells. This physical barrier effect is especially problematic for large molecules like monoclonal antibodies and nanoparticles [62]. Second, the biochemical composition of the ECM influences signaling pathways that enhance cell survival and resistance. Integrins and other ECM receptors on cancer cell surfaces can activate pathways like PI3K/AKT and MAPK, which are linked to survival, proliferation, and resistance to cell death [67].

Cell–cell communication within the TME is also crucial to promote therapeutic resistance. Cancer cells engage in direct communication with stromal, immune, and endothelial cells through cell contacts and secreted factors. For example, CAFs can form gap junctions with cancer cells, facilitating the direct transfer of ions, metabolites, and signaling molecules that enhance survival and resistance [61]. Moreover, exosomes and other extracellular vesicles released by TME cells carry proteins, lipids, and nucleic acids that modulate signaling pathways in recipient cancer cells, further contributing to resistance [63].

### 2.7. Insights into Tumor Microenvironments and Therapy

Recent studies have expanded our understanding of tumor immune microenvironments and their implications for treatment. Key findings include the identification of P2RY6 as an immune marker in lung adenocarcinoma, where its high expression is linked to immune cell infiltration and poor survival outcomes, suggesting its potential as a therapeutic target [69]. Additionally, CXCR6+ CD8 T cells have been identified as pivotal in the immune response to hepatocellular carcinoma, serving as predictive markers for better immunotherapy outcomes [70]. In gastric cancer, ALKBH1 expression has been associated with tumor-associated macrophages, influencing tumor progression and immune evasion, making it a potential target for enhancing anti-tumor immunity [71]. Furthermore, a single-cell multi-omics analysis of desmoplastic small round cell tumors (DSRCTs) has revealed their heterogeneous nature and diverse microenvironmental interactions driving tumor behavior and resistance [72].

Advanced multi-omics techniques have also mapped critical molecular and metabolic pathways involved in immune evasion and tumor progression, providing new insights for therapeutic strategies [73]. Recent research highlights the significance of TP53-related gene signatures in predicting the prognosis and characterizing the TME in bladder cancer, which could guide personalized treatments [74]. Additionally, studies suggest that targeting circadian pathways may improve therapeutic efficacy and survival in certain cancers [75]. Insights from these studies underscore the growing importance of understanding the TME’s role in therapy and resistance, guiding the development of more effective and personalized approaches.

### 2.8. Recent Advances and Future Directions in TME Research

Recent research has brought forth significant discoveries regarding tumor microenvironments (TMEs) and their influence on cancer diagnosis, prognosis, and treatment strategies. Among these findings, the identification of m7G modification patterns linked with TME infiltration in clear cell renal cell carcinoma offers new avenues for therapeutic targeting [76]. Similarly, a three-gene panel has been identified for diagnosing and predicting the prognosis of thyroid papillary carcinoma, providing insights into its immune microenvironment [77]. Additionally, OAS1 has emerged as a vital biomarker across several cancer types, playing a critical role in prognosis, immune response, and therapeutic targeting [78].

Another significant discovery is NNMT’s role as a metabolic regulator in esophageal squamous cell carcinoma, contributing to metastasis and presenting a potential therapeutic target [79]. Moreover, the development of CAMML, a computational tool for immune cell-typing and stemness analysis in single-cell RNA sequencing, has enhanced our understanding of tumor immunology and its impact on therapy [80].

### 2.9. New Therapeutic Strategies and Emerging Biomarkers

Recent research highlights the potential of targeting specific pathways and cell populations within the TME to improve cancer treatment outcomes. For instance, studies have identified the presence of a germinal center-like environment in HLA-DR-positive metastatic melanoma, which could enhance anti-tumor immune responses [81]. In smoking-related bladder cancer, cancer-associated fibroblasts were found to facilitate immune evasion and epithelial–mesenchymal transition (EMT), making them promising therapeutic targets [82]. Additionally, immune and metabolic profiling in lung adenocarcinoma has revealed biomarkers for predicting immunotherapy responses, while the role of CXCL12 in breast cancer progression has been recognized as a potential target for immunotherapy [83,84].

Furthermore, uric acid has been identified as a key factor influencing the immune microenvironment in bladder cancer, suggesting its potential as a prognostic biomarker [85]. Cellular senescence has also been recognized as a critical contributor to cancer progression across various tumor types, and multi-omics analyses from needle core biopsies in glioblastoma have provided new insights into tumor biology and therapeutic targets [86].

## 3. Current Strategies and Therapeutic Approaches

**Figure 6:**
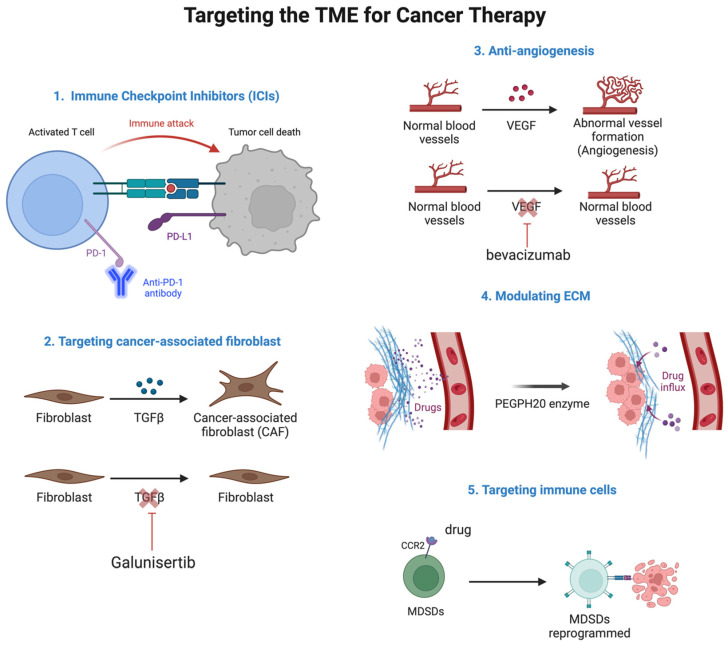
Targeting the TME for cancer therapy. This figure outlines major therapeutic strategies for addressing the tumor microenvironment (TME) in cancer treatment. (1) Immune checkpoint inhibitors (ICIs): anti-PD-1 antibodies block PD-1/PD-L1 interactions, boosting T cell activation and promoting tumor cell death. (2) Targeting cancer-associated fibroblasts (CAFs): galunisertib, a drug that blocks TGF-β signaling, prevents fibroblasts from turning into CAFs, thereby reducing the tumor’s support system. (3) Anti-angiogenesis: bevacizumab targets VEGF to halt abnormal blood vessel formation, restoring normal vasculature and enhancing drug delivery. (4) Modulating ECM: the enzyme PEGPH20 breaks down hyaluronan in the ECM, allowing better drug penetration into the tumor’s core. (5) Targeting immune cells: reprogramming myeloid-derived suppressor cells (MDSCs) via drugs that target the CCR2 pathway can alleviate immunosuppression and restore a robust anti-tumor immune response. These strategies, taken together, offer a comprehensive approach to overcoming the protective barriers of the TME and improving cancer treatment outcomes.

As the understanding of the TME deepens, the development of therapeutic strategies has increasingly focused on targeting the complex interactions between cancer cells and their surrounding environment. The TME plays a critical role in tumor progression, immune evasion, and resistance to conventional therapies, making it a prime target for innovative treatment approaches. Key therapeutic approaches include immune checkpoint inhibitors, targeting CAFs, anti-angiogenic therapies, modulating the ECM, and reprogramming immune cells within the TME (Figure 6).

### 3.1. Immune Checkpoint Inhibitors (ICIs)

Immune checkpoint inhibitors have transformed cancer treatment by focusing on proteins that block T cell activation. Medications like pembrolizumab and nivolumab disrupt PD-1/PD-L1 interactions, enabling the immune system aggressively to target and destroy tumor cells [87]. These inhibitors have shown success in various cancers, including melanoma, lung cancer, and renal cell carcinoma [43].

### 3.2. Targeting CAFs

CAFs play a role in tumor growth and spread by remodeling the ECM and secreting growth factors. Targeting CAFs includes inhibitors of fibroblast activation protein (FAP) and pathways like TGF-β and hedgehog [3]. For example, the TGF-β inhibitor galunisertib has shown promise in curbing CAF activity and boosting chemotherapy effectiveness in pancreatic cancer [88].

### 3.3. Anti-Angiogenic Therapies

Tumors encourage new blood vessel formation through angiogenesis to sustain their growth. Anti-angiogenic therapies like bevacizumab target VEGF signaling to disrupt vascular development. These treatments, combined with chemotherapy, have been used in cancers such as colorectal and ovarian cancer [89].

### 3.4. Modulating the ECM

The ECM offers structural support and controls cell signaling within the TME. Targeting ECM components like collagen and hyaluronan can change the tumor’s physical properties, making it more susceptible to treatment. The enzyme PEGPH20, which degrades hyaluronan, has demonstrated potential to improve drug delivery and survival in pancreatic cancer [90].

### 3.5. Targeting Immune Cells

Reprogramming immune cells within the TME, such as tumor-associated macrophages (TAMs) and myeloid-derived suppressor cells (MDSCs), can shift the environment toward a more anti-tumor response. Drugs targeting CSF-1R and CCR2 pathways are being explored to either reduce or reprogram TAMs [91].

## 4. Applications in Cancer Research and Therapy

The fast-paced advancements in single-cell multi-omics technologies are fundamentally reshaping how we understand cancer biology. These innovations allow scientists to measure multiple molecular aspects like genomics, transcriptomics, proteomics, and metabolomics within individual cells all at once. Such a holistic approach offers new levels of insight into cellular diversity, how tumors interact with their surrounding environment, and the mechanisms that cause resistance to treatment.

One of the significant leaps forward is the combination of single-cell RNA sequencing (scRNA-seq) with other omics layers. Techniques like CITE-seq (Cellular Indexing of Transcriptomes and Epitopes by sequencing) and REAP-seq (RNA Expression and Protein Sequencing) merge transcriptomic and proteomic analysis, allowing researchers to explore both gene expression and protein levels within the same cells [92,93]. These approaches have unveiled new types of cells and states within tumors, deepening our grasp of the functional variety of cancer cells.

In addition, progress in single-cell ATAC-seq (Assay for Transposase-Accessible Chromatin using sequencing) has expanded our understanding of chromatin accessibility at a cellular level, revealing the regulatory blueprints behind gene expression in cancer [94]. The integration of single-cell ATAC-seq with scRNA-seq helps uncover how chromatin structure influences gene activity, shedding light on the role of epigenetic changes in cancer progression.

Spatial transcriptomics (ST) has significantly advanced our understanding of the tumor microenvironment (TME), revealing conserved transcriptional features in tumor cores, such as hypoxia-driven metabolic reprogramming and immune evasion markers, associated with poor prognosis. In contrast, peripheral regions show greater immune infiltration, highlighting spatial immune–tumor interactions that may guide treatment strategies [95]. Additionally, the integration of ST with single-cell RNA sequencing (scRNA-seq) has revealed immune-suppressive and stromal niches, providing insights into tumor–stroma interactions and immune evasion mechanisms that contribute to tumor progression and therapeutic resistance [96].

In glioblastoma, spatial heterogeneity in therapy resistance was linked to increased hypoxia, epithelial-to-mesenchymal transition (EMT), and reduced cytotoxic T cell proximity. Tumor evolution mapping also identified region-specific proliferation and differentiation patterns that traditional sequencing methods could not detect [97]. Together, these studies highlight the transformative potential of ST in identifying critical TME interactions and guiding personalized therapeutic strategies.

Another study highlighted the application of single-cell omics in understanding vasculogenic mimicry (VM) in clear cell renal cell carcinoma (ccRCC). By integrating single-cell RNA sequencing (scRNA-seq) with bulk RNA data, researchers identified a six-gene signature (*L1CAM*, *TEK*, *CLDN4*, *EFNA1*, *SERPINF1*, and *MALAT1*) as key prognostic markers. These genes were linked to tumor invasion, angiogenesis, and immune modulation within the tumor microenvironment (TME). The six-gene risk model effectively stratified patients by survival outcomes and highlighted the potential for combining anti-angiogenic therapies with immunotherapy-targeting VM. This study underscores single-cell omics as a powerful tool for identifying biomarkers and guiding personalized treatment strategies in ccRCC [98].

Another emerging area is single-cell metabolomics, which provides a closer look at the metabolic states of individual cells. With the help of mass spectrometry-based single-cell metabolomics, scientists can now track metabolites within individual cells, directly connecting metabolic shifts to specific cancer cell behaviors [99]. The potential for these single-cell multi-omics technologies in cancer research and treatment is immense.

**Figure 7:**
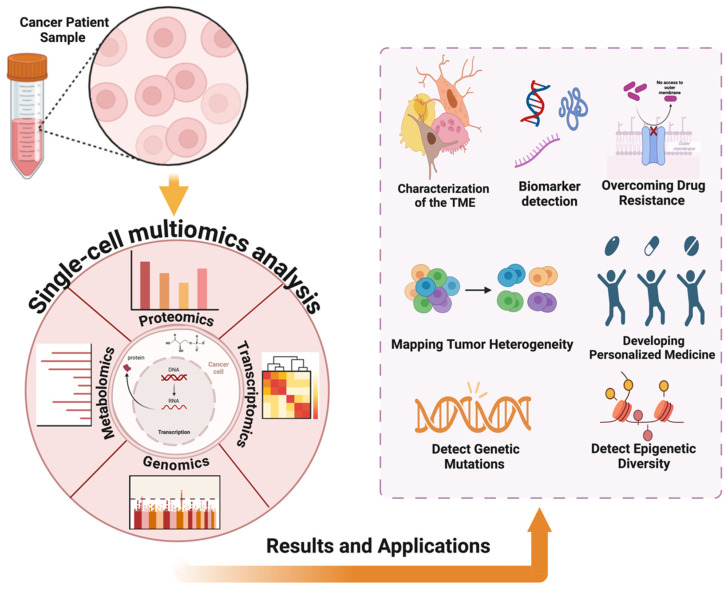
The applications of single-cell multi-omics. This illustration highlights how single-cell multi-omics analysis is revolutionizing cancer research and personalized medicine. By analyzing patient-derived samples with multi-layered approaches (genomics, transcriptomics, proteomics, and metabolomics), researchers gain deep insights into the TME, exposing cellular diversity and the molecular pathways which are driving cancer progression and drug resistance. Key applications include detecting biomarkers, mapping tumor diversity, overcoming treatment resistance, and enabling personalized treatment strategies.

These advanced technologies are crucial for mapping tumor heterogeneity and evolution, identifying distinct cell subpopulations, and tracing their evolutionary paths. This is key to understanding how genetic and epigenetic variations within tumors drive progression and resistance to treatment. By tracking cancer cell evolution, researchers can identify the rise of resistant clones, guiding strategies to prevent or combat resistance [100]. Moreover, single-cell multi-omics allows for an in-depth study of the tumor microenvironment (TME), which includes immune cells, stromal cells, and cancer-associated fibroblasts. Understanding how these cells interact with cancer cells is vital for developing treatments that reshape the TME to boost anti-tumor immune responses. For example, scRNA-seq has helped identify unique immune cell subtypes within tumors, unveiling potential immunotherapy targets. These findings highlight the importance of single-cell multi-omics in driving personalized cancer care and enhancing patient outcomes [101] (Figure 7).

Personalizing cancer therapy with single-cell multi-omics is transforming patient care. By analyzing individual tumors at the single-cell level, doctors can pinpoint the molecular changes driving cancer in each patient, leading to more targeted treatments that minimize resistance and work more effectively. This precision is particularly valuable in rare cancers, where discovering actionable mutations and pathways can inform the use of precise inhibitors [102]. Additionally, single-cell multi-omics is making it possible to detect early warning signs and predict cancer outcomes. Biomarkers identified in blood or other bodily fluids provide a non-invasive way to catch the disease early and monitor its progress over time. For instance, analyzing circulating tumor cells (CTCs) using these cutting-edge techniques reveals signatures linked to metastasis. These breakthroughs emphasize the game-changing potential of single-cell multi-omics in enhancing personalized cancer treatment and making early detection more accessible [103].

The ongoing development and application of single-cell multi-omics technologies will shape the future of cancer research and therapy. These approaches offer a comprehensive view of cancer biology at unparalleled resolution, opening up new pathways for personalized and targeted treatments. As these technologies become more widely accessible and integrated, their impact on transforming cancer care and improving patient outcomes will only increase.

Recent advancements in understanding the TME using single-cell multi-omics have greatly deepened our knowledge of cancer progression and treatment resistance. The TME, made up of cancer cells, stromal cells, immune cells, and the extracellular matrix, plays a pivotal role in shaping tumor behavior and response to therapies. Key findings highlight the cellular diversity within the TME, with single-cell sequencing uncovering varied populations of cancer-associated fibroblasts, immune cells like T cells and macrophages, and endothelial cells, each displaying distinct functional states that contribute differently to tumor growth and treatment responses [104]. Moreover, single-cell transcriptomics has provided detailed insights into the immune landscape of tumors, identifying specific immune cell subsets and their interactions with cancer cells, which illuminate immune evasion strategies. For instance, exhausted T cells, characterized by the expression of inhibitory receptors like PD-1 and CTLA-4, are prevalent in the TME and are associated with a poor prognosis. Additionally, tracking therapy-induced changes at the single-cell level has offered valuable clues about how cancer cells adapt to treatments, aiding the design of combination therapies to prevent or delay resistance. Fresh tissue multi-omics profiling in conventional chondrosarcoma has helped classify immune subtypes and identify potential candidates for immunotherapy, enhancing treatment approaches [8].

Single-cell metabolomics has revealed the metabolic diversity within tumors, demonstrating how different metabolic profiles in cancer and stromal cells contribute to the overall reprogramming of the TME. This reprogramming supports cancer cell growth and survival in low-oxygen environments, and plays a role in resistance to therapy [9].

## 5. Clinical Trials

This section reviews a series of studies employing advanced multi-omics techniques, such as single-cell RNA sequencing, spatial transcriptomics, and comprehensive genomic analyses, to investigate various cancers. These studies aim to identify novel biomarkers, understand immune landscape dynamics, and develop personalized treatment strategies, ultimately enhancing patient outcomes. By dissecting the molecular and cellular intricacies of different cancers, these investigations pave the way for more precise and effective therapeutic interventions. There are several clinical trials focusing on the role of the tumor microenvironment (TME) in cancer progression and therapeutic resistance, with insights from single-cell multi-omics technologies (Table 1).

## 6. Exploring New Cancer Treatment Avenues via Multi-Omics Analysis

Research has identified a protective barrier within liver cancer tumors, made up of specific immune and support cells. This barrier prevents immune cells from reaching the tumor, reducing the effectiveness of immunotherapy [118]. Recent breakthroughs have uncovered ways to disrupt this barrier, improving the immunotherapy response in early trials. Simultaneously, a novel type of cell death called cuproptosis has been connected to immunosuppressive environments in several cancers [119]. Cuproptosis-related genes have been linked to poor outcomes and immune evasion, suggesting that therapies targeting both the physical tumor barrier and cuproptosis could be promising [120]. Additionally, the single-cell analysis of testicular and pancreatic cancers has uncovered unique immune cell groups with signs of exhaustion, offering clues to combat tumor progression. The power of single-cell technologies lies in revealing the complex tumor environment, aiding the creation of new treatments [121].

Studies in pancreatic cancer have highlighted the diversity within tumor-associated neutrophils, with some subtypes driving tumor growth by increasing glycolysis—a process fueled by a specific transcription factor. These findings offer potential therapeutic targets [122].

Despite progress in treating hepatocellular carcinoma (HCC) and clear cell renal cell carcinoma (ccRCC), challenges remain. HCC’s immunosuppressive environment, with exhausted T cells and suppressive macrophages, continues to resist treatment efforts. In ccRCC, although models have been developed to predict outcomes, translating these insights into real-world benefits is difficult due to the tumor’s genetic and cellular complexity [37].

Although single-cell technologies have made significant strides in mapping tumor cells and their environments, there is still much to learn about how these elements interact to drive cancer growth. While we now have potential molecular targets and a clearer understanding of cell populations linked to tumor progression, the exact mechanisms are still murky. This gap in knowledge hinders the development of more effective treatments.

Emerging insights from multi-omics studies emphasize the intricate relationships between tumor cells, immune cells, and the surrounding environment. For instance, targeting the molecular driver SERPINE2 in renal cell carcinoma, understanding the role of tumor stromal cells in colorectal cancer, and mapping ligand–receptor networks in squamous cell carcinoma all underscore the importance of the tumor environment in cancer progression and immune escape [123].

A comprehensive understanding of T cell exhaustion across cancers remains a challenge. While distinct profiles linked to patient outcomes have been identified, the specific mechanisms driving immune escape are still unclear. This ongoing research highlights the need for a deeper exploration into the interactions between tumor cells, immune cells, and their environment [124].

## 7. Integrating Multi-Omics into Cancer Therapeutic Strategies

Recent studies have spotlighted various factors that are pivotal in cancer development and treatment. For example, research has identified LSM1 as a key player in breast cancer metabolism and macrophage infiltration in tumors, indicating its therapeutic potential [125]. Similarly, CLIC1 has been highlighted as both a promising therapeutic target and a biomarker in gliomas. Novel diagnostic markers and inflammatory profiles have been identified in renal angiomyolipoma linked to tuberous sclerosis complex, paving the way for more precise therapeutic approaches [126]. Regulatory T cells are also shown to affect the tumor immune environment and responses to immunotherapy in triple-negative breast cancer, suggesting their potential as therapeutic targets [127,128]. Furthermore, Apolipoprotein E’s role in alternative splicing and its influence on the immune microenvironment in kidney renal clear cell carcinoma have positioned it as a promising therapeutic target [129].

Advancements in our understanding of tumor microenvironments and strategies for treating diverse cancers have also been substantial [130]. For instance, certain tumor microenvironment-related genes have emerged as predictors of prognosis in pancreatic cancer. Additionally, a classifier focusing on macrophage differentiation and multi-omics data has been developed to enhance treatment strategies for papillary thyroid cancer [131]. The role of IMMT in breast cancer is now recognized, emphasizing its value as a potential theragnostic marker [132]. Another noteworthy development is a risk model centered on interferon regulatory factors that guides immunotherapy for clear cell renal carcinoma [133]. Recent studies have also explored immunogenic cell death pathways in gastric adenocarcinoma and identified PFKFB3 as a major factor in sunitinib resistance for papillary renal cell carcinoma, offering it as a potential target for improved treatment responses [134,135].

New research has greatly enhanced our grasp of cancer biology and treatment options. Single-cell profiling, for instance, has shed light on the complex biology and environment within primary brain tumors, guiding more targeted treatment approaches [136]. Additionally, a novel classifier based on G protein-coupled receptors and the tumor microenvironment has been developed for predicting survival and immunotherapy responses in melanoma [137]. Moreover, the roles of toll-like receptors across different cancers have been emphasized for their relevance in tumor progression and potential therapeutic avenues [138]. Functional state-based molecular subtypes of gastric cancer have been identified, supporting the adoption of personalized treatment approaches [139]. Tools like GBMdeconvoluteR have also been introduced, offering a more accurate analysis of neoplastic and immune cell proportions in glioblastoma [140]. Through multi-omics analyses, basement membrane genes have been identified as novel biomarkers and therapeutic targets in uveal melanoma [141].

Several studies have revealed significant findings with potential implications for cancer prognosis and treatment. For instance, FABP5 has emerged as a critical gene in gliomas, linked to liquid–liquid phase separation and suggested as a prognostic marker and therapeutic target [142]. AIM2 inflammasomes have been highlighted in various cancers as potential targets for immunotherapy [143]. Machine learning models have been developed to assess perineural invasion risks in head and neck squamous cell carcinoma and to identify macrophage-related therapeutic modules in colorectal cancer [144]. CD73 has also surfaced as a prognostic biomarker and potential immunotherapy target in intrahepatic cholangiocarcinoma [145]. Insights from advanced imaging and multi-omics integration have unveiled cell type-specific features in breast cancer and crucial GPCR gene markers in lung adenocarcinoma, paving the way for more effective targeted therapies [146,147]. Molecular characteristics of parthanatos in gastric cancer and a gene enrichment score related to glycerolipid metabolism in colon cancer have also been linked to prognosis, offering new therapeutic pathways [148,149]. Additionally, studies on arginine biosynthesis genes point to new strategies for overcoming tumor immune evasion and therapy resistance [150].

The understanding of cancer biology and possible therapeutic interventions has been further enriched by recent findings across several cancer types. For instance, MYC signaling in lung adenocarcinoma has been identified as a key driver of tumor progression and a viable therapeutic target [151]. In metastatic melanoma, pyroptosis-related markers offer new possibilities for enhancing immunotherapy outcomes [152]. Macrophage-specific cathepsin in clear cell renal cell carcinoma has also been identified as a prognostic marker [153]. Studies on colorectal cancer have shown that precursor exhausted CD8+ T cells are linked to survival rates and responses to immunotherapy [154]. Additionally, a prognostic signature for pancreatic adenocarcinoma based on ubiquitination-related mRNA and lncRNA has been proposed [155]. Single-cell and multi-omics approaches have further revealed how Euphorbiae Humifusae Herba induces mitochondrial dysfunction in non-small-cell lung cancer, while gamma-delta T lymphocytes have shown potential for improving the tumor microenvironment and immunotherapy outcomes in cervical cancer [156,157]. Moreover, a multi-omics pan-cancer analysis has identified CREB5 as a key biomarker for prognosis and immunotherapy responses in glioma [158].

Recent studies have highlighted key insights into cancer progression and therapy. Glycogen synthase 1 has been identified as a crucial target for inducing disulfidptosis in triple-negative breast cancer, pointing to new therapeutic possibilities [159]. The heterogeneity of macrophages in glioma patients has been linked to tumor progression and therapy outcomes [160]. Transcriptomics and deep multiplex imaging have mapped bone marrow metastasis in neuroblastoma, uncovering potential therapeutic targets [161]. T-bet has also been identified as a suppressor of malignant B cell proliferation in chronic lymphocytic leukemia, suggesting its therapeutic relevance [162]. Dysregulated neurogenesis-related genes in the tumor immune microenvironment have been proposed as potential biomarkers and therapeutic targets [163]. Additionally, pyroptosis-related markers have been identified as critical for optimizing neoadjuvant immunotherapy in gastric cancer, which could enhance treatment outcomes [164].

Recent research continues to shed light on the intricacies of cancer progression and novel therapeutic strategies. For example, Glycogen synthase kinase 1 has been identified as a critical target for inducing disulfidptosis in triple-negative breast cancer, offering new potential avenues for treatment [159]. Additionally, the diversity of macrophages in glioma patients has been linked to differences in tumor progression and therapeutic responses [160]. Transcriptomics and deep multiplex imaging have also been employed to map bone marrow metastasis in neuroblastoma, revealing promising therapeutic targets [161]. In chronic lymphocytic leukemia, T-bet has been shown to suppress malignant B cell proliferation, positioning it as a potential therapeutic target [162]. Dysregulation of neurogenesis-related genes within the tumor immune microenvironment has emerged as a promising area for the identification of biomarkers and therapeutic targets [163]. Lastly, key pyroptosis-related markers have been identified as essential for optimizing neoadjuvant immunotherapy in gastric cancer, with the potential to improve treatment outcomes [164].

## 8. Prognostic Biomarkers and Therapeutic Targets

Recent breakthroughs have significantly refined cancer diagnosis and treatment methods. For instance, novel approaches in lung adenocarcinoma uncovered immune suppression subtypes, leading to the development of a prognostic signature that deepens the understanding of tumor progression [165]. Similarly, in osteosarcoma, an m7G-related signature now helps predict both the prognosis and response to immunotherapy, paving the way for more personalized treatment strategies [166]. Moreover, the creation of a Precancer Atlas, designed to detect early tissue changes, promises to enhance preventive measures and early intervention efforts [167]. Notably, a prognostic signature based on necroptosis-related genes has been introduced for cervical cancer, offering innovative patient stratification techniques [168]. Further research on hepatocellular carcinoma explored the relationship between disulfidptosis and the immune microenvironment, pointing to fresh immunotherapeutic strategies [169]. Additionally, the significance of C1QC+ and SPP1+ tumor-associated macrophages in cervical cancer was highlighted, and single-cell analyses revealed evolutionary mimicry across breast cancer subtypes, providing a new lens for classification and treatment [170,171].

Recent findings also underscore a deeper grasp of cancer progression and emerging therapeutic targets. A variety of genes and molecular mechanisms that shape prognosis, immune microenvironments, and drug responsiveness have been uncovered across several cancer types. For instance, the ADAMTS gene family, galectins, and FXYD5 have been spotlighted as critical biomarkers, while research notes the co-regulation of tumor-stromal heterogeneity by MUC16 and TP53 in pancreatic adenocarcinoma [172,173,174]. Additionally, immune cell-related gene expression models now provide deeper insights into tumor immune dynamics, while the role of FTO in promoting tumor neovascularization via m6A modification removal has been highlighted [175,176]. For glioblastoma, CpG island methylation patterns present a novel classification system, revealing potential therapeutic targets for improved outcomes [177].

Diverse therapeutic strategies continue to be explored across different cancer types. For instance, research has delved into genes and pathways associated with vasculogenic mimicry, immune microenvironments, and cancer-associated fibroblasts, all aimed at stifling cancer progression and enhancing treatment outcomes [178]. Systems pharmacology approaches have also emerged as promising methods to boost the effectiveness of PD-1/PD-L1 blockade, a key cancer immunotherapy [179]. Additionally, cuproptosis-related genes in hepatocellular carcinoma and variations in surface protein abundance in metastatic melanoma have shed light on new avenues for targeted therapies [180]. The role of the CCN family in regulating cell interactions and immune evasion in glioma, along with the impact of JAG1 on immune microenvironments and resistance to immunotherapy in lung adenocarcinoma, have been identified as promising areas for targeted treatment [181,182]. Moreover, antigen-specific T cell receptor clusters across various cancers present opportunities for personalized immunotherapy approaches [183]. These advancements collectively enhance our understanding of cancer biology and open new pathways for more effective therapeutic solutions.

Further investigations into prognostic biomarkers and therapeutic targets have yielded valuable insights across multiple cancer types. In gastric cancer, for instance, YWHAE has been flagged as a key gene in ferroptosis, offering new therapeutic possibilities [184]. Similarly, in head and neck squamous cell carcinoma, SLC25A17 emerged as a novel biomarker for forecasting prognosis and immune microenvironment changes [185]. A multi-omics profiling of fresh tissues in conventional chondrosarcoma classified immune subtypes, informing immunotherapy candidates and treatment strategies [186]. A polyamine gene expression score was also developed to predict the prognosis and treatment response in clear cell renal cell carcinoma, pointing to personalized therapy options [187]. Furthermore, inhibiting PI3K/mTOR has been found to remodel the tumor microenvironment and sensitize pS6high uterine leiomyosarcoma to PD-1 blockade, suggesting new therapeutic approaches [188]. Different head and neck squamous cell carcinoma subtypes were also identified based on mononuclear phagocyte system-related multi-omics features, offering insights into prognosis and treatment responses [189]. These approaches have been applied to investigate immune-checkpoint blockade (ICB) therapies in human cancers. A study utilizing previously published single-cell transcriptomics, epigenomics, and TCR data revealed that tumor-reactive T cells responded differently to ICB across various tumor types. Notably, the presence of CXCL13+ CD8+ T cells was associated with a favorable response to ICB [190].

Single-cell multi-omics has been utilized to evaluate chimeric antigen receptor (CAR) T cell therapy in acute lymphoblastic leukemia by integrating transcriptomics and proteomics data. This approach identified molecular differences underlying complete remission, non-responsiveness, and relapse. The analysis highlighted TH2 cell pathways and associated genes as potential targets for sustaining long-term remission, particularly over five years, following CAR T cell immunotherapy [191]. Lastly, machine learning has been employed to study regulated cell death pathways in glioma, proving effective in predicting both patient prognosis and immunotherapy outcomes [192].

The discovery of various prognostic biomarkers and therapeutic targets continues to advance cancer research. For example, one study identified B7-H3 as a prognostic biomarker in head and neck squamous cell carcinoma, correlating it with the response to immune checkpoint blockade therapies [193]. Another highlighted aldehyde dehydrogenase 2 as a key player in mediating interactions between Treg-mediated immunosuppression and hepatocellular carcinoma, offering fresh targets for therapy [194]. Furthermore, the reciprocal interaction between Th17 cells and mesothelial cells, promoting adhesion in ovarian cancer metastasis, hints at new treatment options [195]. PANoptosis-related markers in glioma also provide critical insights into prognosis and tumor microenvironment understanding. Additional studies found that ALG3 suppresses CD8+ T cell infiltration by inhibiting chemokine secretion, impacting the efficacy of 5-fluorouracil in cancer therapy [196]. Another study formulated a hypoxia prognostic signature for glioblastoma multiforme using bulk and single-cell RNA-seq data, refining patient stratification methods for treatment [197]. Finally, the mitochondrial AAA protease gene has been identified as a prognostic marker linked to immune infiltration in ovarian cancer, revealing potential new treatment paths [198].

## 9. Advances in Multi-Omics and Personalized Cancer Therapy

Recent strides in cancer research have revealed the vital role that genetic and molecular markers play in refining cancer prognosis, immune interactions, and therapeutic outcomes across a variety of cancers. For instance, a combination of single-cell and bulk RNA sequencing has pinpointed a gene signature linked to neoadjuvant chemotherapy in breast cancer, which offers a way to predict survival and treatment response, thereby paving the way for more tailored treatment strategies [199]. Multi-omics investigations also uncovered that CD276 stands as a significant prognostic marker in several cancers, including glioblastoma, shedding light on new possibilities for targeted therapies [200]. In colorectal cancer, studies revealed that CTSB+ macrophages impede memory immune responses in liver metastases, offering potential therapeutic avenues to enhance immune responses [201]. Moreover, integrating multi-omics with machine learning has revealed disulfidptosis patterns in lung adenocarcinoma, which could predict immunotherapy outcomes and assist in personalizing treatment plans [202]. Collectively, these findings underscore the growing significance of sophisticated genetic and molecular analyses in shaping individualized therapies and boosting patient outcomes across various cancer types.

Continued exploration has further uncovered key biomarkers and cellular behaviors in different cancers, offering critical insights for personalized treatment approaches. In pancreatic cancer, TLR2 has been identified as a prognostic marker, highlighting the variability in NETosis that could guide customized treatments [203]. High-resolution transcriptomics in lung adenocarcinoma identified a cell subtype marked by the expression of CXCL13, EPSTI1, and CDK1, suggesting possible therapeutic targets [204]. For early-stage endometrial cancer, a newly discovered oncogenic cluster with low TRAP1 and CAMSAP3 expression was linked to more aggressive behavior and a poorer prognosis [205]. These collective insights emphasize the value of integrating multi-omics data to deepen our understanding of cancer biology and enhance patient-specific therapeutic strategies.

Research has also highlighted pivotal findings in different cancer types through advanced genetic and molecular approaches. Hypoxia-related genes have emerged as key factors in TACE-refractory hepatocellular carcinoma, impacting the prognosis, immune characteristics, and drug resistance [206]. In lung adenocarcinoma, a gene signature linked to cuproptosis and anoikis was developed to predict the prognosis and immune infiltration [207]. Meanwhile, in lower-grade glioma, a mitochondrial RNA modification-based signature was identified, improving patient stratification and prognosis prediction [208]. In cervical cancer, hypoxia subtypes and the immunosuppressive factor S100A2 were recognized as new therapeutic targets [209]. Moreover, an integrative multi-omics analysis, coupled with machine learning, revealed significant GPCR gene features in lung adenocarcinoma, offering new perspectives for targeted therapies. Single-cell profiling methods have also shed light on the biology and microenvironment of primary brain tumors, aiding in the development of more precise therapies [146]. These findings collectively highlight the essential role of multi-omics in personalizing cancer treatments and enhancing patient care.

Further insights from multi-omics analyses continue to shape our understanding of cancer biology, prognosis, and treatment strategies. For example, fresh tissue profiling in conventional chondrosarcoma helped classify immune subtypes and identified candidates for immunotherapy, presenting new therapeutic possibilities [186]. In head and neck squamous cell carcinoma, B7-H3 emerged as a new prognostic marker and predictor for immune checkpoint blockade response, offering fresh therapeutic targets [193]. Aldehyde dehydrogenase 2 was found to be a key factor in the immunosuppressive environment of hepatocellular carcinoma, suggesting new therapeutic interventions [194]. In glioma, predictors linked to PANoptosis were identified, providing insights into prognosis and tumor microenvironment characteristics, thereby guiding personalized treatment approaches [210]. These studies collectively underscore the importance of multi-omics techniques in pinpointing biomarkers and crafting targeted therapies that ultimately improve cancer treatment and patient outcomes.

Genomics, transcriptomics, proteomics, and metabolomics are pivotal components of personalized medicine, each contributing to more precise, individualized healthcare. Genomics, through advances like next-generation sequencing (NGS), enables detailed genetic analysis to identify disease-associated mutations, guiding early detection and tailored therapies, particularly in cancer through biomarkers such as BRCA1 and BRCA2 mutations. CRISPR gene-editing technologies further enhance personalized medicine by allowing precise DNA modifications, offering potential cures for genetic disorders [211]. Transcriptomics, focusing on RNA transcripts, provides insights into gene expression patterns and their role in disease. RNA sequencing (RNA-seq) identifies molecular signatures that improve disease diagnosis, optimize treatment strategies, and uncover activated or suppressed biological pathways. This integration of genomics and transcriptomics enables the development of targeted therapies that minimize side effects, especially in cancer, by distinguishing subtypes and predicting disease progression [212,213].

Proteomics and metabolomics complement these technologies by offering insights into proteins and metabolites, respectively, providing a comprehensive view of biological processes. Proteomics, using techniques like mass spectrometry, identifies disease-specific protein expression profiles, aiding in early diagnosis, therapy selection, and predicting drug responses. It also identifies proteins involved in drug resistance, guiding personalized therapeutic approaches [214]. Metabolomics focuses on metabolites, which reflect the physiological state of an organism, offering biomarkers for early disease detection and personalized treatment strategies. It helps monitor patient responses to therapy, adjusting treatments as necessary, and uncovers new therapeutic targets. When integrated with genomics, transcriptomics, and other omics technologies, metabolomics enhances the understanding of health and disease, improving the precision and effectiveness of personalized medicine [211].

Finally, comprehensive multi-omics analyses have brought forth significant revelations in cancer prognosis and treatment. For instance, ALG3 was found to hinder CD8+ T cell infiltration by suppressing chemokine secretion, affecting 5-fluorouracil sensitivity in various cancers and presenting novel therapeutic targets [196]. In glioblastoma multiforme, a hypoxia prognostic signature was crafted using bulk and single-cell RNA sequencing, refining the prediction of patient outcomes and treatment responses [197]. Additionally, a signature associated with cuproptosis in colorectal cancer was identified, offering a means to predict prognosis and immunotherapy effectiveness, further enhancing personalized treatment plans [215]. These collective insights emphasize the pivotal role of multi-omics approaches in refining cancer prognosis, guiding therapeutic choices, and developing more targeted treatments.

## 10. Limitations of Single-Cell Technologies

Although single-cell technologies have greatly enhanced our understanding of tumor heterogeneity, several limitations persist, such as challenges related to sensitivity, scalability, and accuracy. Overcoming these issues requires ongoing technological improvements or the integration of complementary approaches [216]. Additionally, many single-cell methods rely on dissociating cells, which limits the ability to analyze the spatial architecture of tumor tissues. The development of spatial transcriptomics (ST) provides a partial solution to this limitation, but the resolution of current ST platforms remains relatively low, with capture spots often containing multiple cells [217]. Furthermore, the process of preparing single-cell suspensions can disrupt the transcriptome and proteome. Many single-cell RNA sequencing (scRNA-seq) techniques are also restricted to detecting only protein-coding genes by capturing polyA RNAs, which means non-coding genes are excluded. Moreover, interpreting data from single-cell omics is challenging, as it relies heavily on bioinformatics tools, each with its own set of strengths and limitations. For example, the identification of cell types within a tumor can vary depending on the parameters used in different algorithms [218].

Moreover, tumor heterogeneity remains a significant obstacle, as multi-omics methods cannot fully capture the complexities of diverse tumor populations. Temporal dynamics, which are crucial for understanding cancer initiation, progression, and therapy responses, may be overlooked [219]. Furthermore, interpreting results requires careful consideration of the limitations of individual technologies used in multi-omics studies. Validating the biological relevance of findings often necessitates additional functional studies. Ethical and legal concerns, such as patient privacy, data sharing, and compliance with ethical standards, also pose barriers to multi-omics research [220]. Finally, the high costs associated with data acquisition, sample processing, and specialized equipment make these investigations financially burdensome, potentially limiting their accessibility and implementation [218].

## 11. Future Directions

Tracking therapy-induced changes at the single-cell level offers key insights into how cancer cells adapt to treatments. This knowledge can be used to create combination therapies designed to prevent or delay resistance. For instance, single-cell RNA sequencing has been a valuable tool in studying how chemotherapy affects tumor cell populations. Through this approach, researchers have identified resistance mechanisms and potential combination treatments. In conventional chondrosarcoma, fresh tissue multi-omics profiling has classified immune subtypes and highlighted potential immunotherapy candidates, helping to refine treatment strategies. Similarly, in head and neck squamous cell carcinoma, B7-H3 has emerged as a promising prognostic biomarker and an indicator for predicting responses to immune checkpoint blockade, thus opening up new therapeutic possibilities. Aldehyde dehydrogenase 2 has been found to play a pivotal role in the immunosuppressive environment of hepatocellular carcinoma, pointing to potential new treatment approaches. Additionally, PANoptosis-related indicators have been identified for prognosis and tumor microenvironment characteristics in glioma, providing a pathway toward personalized therapies.

The field of single-cell multi-omics in cancer research is advancing rapidly, bringing several exciting possibilities. The development of computational tools and methodologies that integrate single-cell genomic, transcriptomic, proteomic, and metabolomic data will offer a more comprehensive understanding of the tumor microenvironment (TME), helping uncover new interactions and regulatory networks that drive cancer progression [7]. Longitudinal studies using single-cell technologies can observe the dynamic changes in the TME over time and in response to treatment, enabling the identification of early resistance biomarkers and facilitating the design of adaptive therapies. Furthermore, combining single cell sequencing with spatial transcriptomics can map out how cells are spatially organized within the TME, potentially revealing new therapeutic targets and enhancing treatment precision [13].

Integrating single-cell multi-omics with PROTAC (Proteolysis-Targeting Chimera) probe technology offers a powerful strategy for advancing cancer drug discovery. Single-cell multi-omics reveals cell-specific vulnerabilities within the tumor microenvironment (TME), while PROTAC probes selectively degrade key proteins, including previously undruggable targets, via the ubiquitin–proteasome system. This synergy enables the identification and validation of novel therapeutic targets, mechanistic insights into TME-driven processes like immune evasion and angiogenesis, and the development of personalized treatments tailored to patient-specific TME profiles. Together, these technologies provide a precise and innovative approach to overcoming resistance and improving cancer therapies [221].

As single-cell multi-omics technologies continue to evolve, their potential in cancer research and treatment becomes even more promising. Leveraging these techniques to craft personalized treatment plans based on an individual’s unique TME can optimize the effectiveness of therapy while minimizing side effects [14]. As these technologies become more widely available and integrated, they could significantly reshape cancer care by offering new paths for personalized and targeted therapies [6]. The deep, detailed understanding of cancer biology made possible by these multi-omics approaches highlights their importance in revolutionizing cancer treatment.

## 12. Conclusions

Recent advancements in single-cell multi-omics technologies mark a breakthrough in cancer research, delivering a level of insight into the tumor microenvironment (TME) that was previously unattainable. By combining genomic, transcriptomic, proteomic, and metabolomic data at the single-cell level, researchers are better equipped to explore the complex cellular and molecular diversity within tumors. This approach sheds light on new mechanisms of immune evasion, metabolic shifts, and resistance to therapies. Not only do these findings deepen our understanding of cancer biology, but they also offer a path toward crafting more precise, individualized treatment strategies targeting distinct cellular subgroups and pathways in the TME. As these tools evolve, pairing them with spatial transcriptomics, longitudinal studies, and cutting-edge computational models will refine our ability to track dynamic TME shifts, spot early signs of resistance, and fine-tune therapeutic approaches. The path forward in precision oncology lies in harnessing single-cell multi-omics data to tailor treatments according to unique tumor profiles, which will ultimately enhance patient outcomes and reduce side effects. The ongoing pursuit and implementation of these techniques promise to revolutionize cancer therapy, ushering in the next chapter of personalized medicine and offering new solutions for tackling therapeutic resistance and tumor complexity.

## Figures and Tables

**Table 1:** Clinical trials employing single-cell multi-omics approach to treat different types of cancer.

Study Title	Reg. No.	Country	Pts. No.	Cancer Type	Description	Ref.
Single-cell RNA sequencing reveals the tumor microenvironment and facilitates strategic choices to circumvent treatment failure in a chemorefractory bladder cancer patient.	DOI: 10.1186/s13073-020-00741-6	Korea	1	Bladder cancer	This study uses single-cell RNA sequencing to analyze the tumor microenvironment of a chemorefractory bladder cancer patient, aiming to identify strategic choices to overcome treatment failure	[105]
Mechanism of Response to IMFINZI Neoadjuvant Therapy in Non-small Cell Lung Cancer Patients Based on Multiple-omics Models.	NCT04646837	China	20	Non-small lung cancer	This single-center, exploratory study examines the impact of neoadjuvant PD-1 monoclonal antibody therapy by analyzing blood and tumor samples before and after treatment.	[106]
Analysis of the Microenvironment of Lung Cancer and Exploration of the Mechanism of Resistance to Immunotherapy	NCT05636605	China	200	Lung cancer	Investigators used multi-omics analyses, including genomics, proteomics, single-cell RNA sequencing, and spatial transcriptomics on tumor tissue and blood. The goal was to analyze tumor heterogeneity, map the lung cancer microenvironment, and explore mechanisms of sensitivity and resistance to anti-PD1/PD-L1 antibodies.	[107]
Improving Personalised Glioblastoma Care by Stem Cell Analysis, Omics (Including Immunomics) and Artificial Intelligence Approaches	NCT05941234	Italy	120	Glioblastoma	This study aimed to integrate in-depth multi-omics with clinical data to discover immune markers in glioblastoma patients. It considered age and sex differences, predicted prognosis, defined key life/environmental factors, and guided AI-driven personalized treatments to improve care and quality of life.	[108]
Multi-omics Tumor Evolution Model of NSCLC	NCT05352035	China	300	Non-small cell lung cancer	The purpose of this study was to determine the evolutionary mechanism of early-stage non-small cell lung cancer and establish an accurate prognostic model and a recurrence monitoring system using multi-omics analysis, which can be helpful for the individual and whole management of lung cancer patients and improve the overall prognosis.	[109]
Multi-omics Characterization of Pancreatic Neuroendocrine Tumors and Carcinomas	NCT05234450	France	300	Pancreatic cancer	Researchers aimed to identify distinct subgroups within pancreatic neuroendocrine tumors and carcinomas, using integrated multi-omics analysis. Their approach involved next-generation sequencing methods like RNAseq, along with the MCP Counter tool to quantify eight immune cell populations in TME.	[110]
Investigation of Tumor Microenvironment After CRPC Along With Before and After Neoadjuvant Therapy for Prostate Cancer	NCT05522907	China	1000	Prostate cancer	In a retrospective study, investigators perform multi-omics analysis (including whole exome, RNAseq), immune cell characterization, and biopsy samples from prostate cancer primary biopsy, ADT neoadjuvant, and CRPC biopsy samples in the biobank.	[111]
Neuroendocrine Neoplasm Based on Multi-omics Integrated Analysis	NCT04931446	China	200	Neuroendocrine neoplasm	This project analyzed the molecular biological characteristics of NEN based on multi-omics, developing an exclusive NEN multi-omics big data platform, and carrying out molecular subtypes and potential targets predictions to improve the therapeutic effect of neuroendocrine tumors.	[112]
Single-cell RNA Sequencing Resolves the Regulatory Role of HBV on the Hepatocellular Carcinoma Immune Microenvironment	NCT05677724	China	20	Primary liver cancer and HBV	With the help of single-cell sequencing technology, this study focused on elucidating the influence of HBV-induced hepatocellular carcinoma cell metabolic changes on microenvironment remodeling. This study provides a more accurate diagnosis and treatment method for HBV-induced hepatocellular carcinoma.	[113]
Tumor Microenvironment in Ovarian Cancer (MICO)	NCT06272240	Italy	50	Ovarian cancer	This research aimed to create ovarian cancer organoids to explore interactions and molecular pathways between tumor cells, immune cells, and the local microbiota in a controlled laboratory setting.	[114]
Deep, Multi-omics Phenotyping to Predict Response, Resistance and Recurrence to Adjuvant Atezolizumab Plus Bevacizumab in Resected Hepatocellular Carcinoma (EMPHASIS)	NCT05516628	Singapore	30	Hepatocellular carcinoma	This study explored the molecular complexity of hepatocellular carcinoma (HCC) to find predictive biomarkers for personalized therapies. Through two clinical studies with Atezolizumab plus Bevacizumab and Yttrium-90, it collected longitudinal biosamples. The goal was to understand the tumor microenvironment, biomarker co-localization, and their impact on treatment response and recurrence in HCC.	[115]
Comparison of the Breast Tumor Microenvironment	NCT03165487	United States	30	Triple-negative breast cancer	The project aimed to define a molecular profile of tumor stroma using “normal” adjacent breast tissue collected before and after intraoperative radiation therapy during breast conserving surgery. This research specifically focused on patients with luminal A and triple-negative breast cancer, where IORT is a standard treatment.	[116]
Deciphering the Immune Microenvironment at the Forefront of Tumor Aggressiveness by Constructing a Regulatory Network with Single-Cell and Spatial Transcriptomic Data.	DOI: 10.3390/genes15010100	China	-	Breast cancer	This study constructed intercellular gene regulatory networks using single-cell RNA sequencing and spatial transcriptomics data to understand the immune microenvironment at the invasive front of ER-positive breast cancer.	[117]

## Data Availability

All data are provided in the paper.

## Abbreviations

ATAC-seq: Assay for Transposase-Accessible Chromatin, BRAF: B-Raf proto-oncogene, serine/threonine kinase, BRCA1: Breast Cancer gene 1, CAFs: Cancer associated fibroblasts, CAR-T: chimeric antigen receptor T cell, ccRCC: Clear cell renal cell carcinoma, CDK1: Cyclin-dependent kinase 1, CITE-seq: Cellular Indexing of Transcriptomes and Epitopes by sequencing, CSCs: Cancer stem cells, CTCs: Circulating tumor cells, CTLS: Cytotoxic T lymphocytes, CXCL12: C-X-C motif chemokine 12, DSRCTs: Desmoplastic small round cell tumors, ECM: Extracellular matrix, EGFR: Epidermal growth factor receptor, EMT: Epithelial-mesenchymal transition, EPSTI1: Epithelial Stromal Interaction, GPCR: G-protein-coupled receptors, HBV: Hepatitis B virus, HCC: hepatocellular carcinoma, HER2: Human epidermal growth factor receptor-2, HGF: Hepatocyte growth factor, HIFs: Hypoxia-inducible factors, iCAFs: Inflammatory CAFs, ICIs: Immune checkpoint inhibitors, IL-1: Interleukin-1, lncRNA: Long non-coding RNA, ATAC-seq: Assay for Transposase-Accessible Chromatin, MAPK: Mitogen-activated protein kinase, MDSCs: Myeloid-derived suppressor cells, MET: Mesenchymal-epithelial transition, MICO: TME in Ovarian Cancer, MMPs: metalloproteinases, myCAFs: Myofibroblastic CAFs, NK: Natural killer, NNMT: Nicotinamide N-Methyltransferase, NO: Nitric oxide, NSCLC: Non-small cell lung cancer, OXPHOS: Oxidative phosphorylation, PD-1: Programmed cell death-1 receptor, PD-L1: Programmed death-1 ligand 1, REAP-seq: RNA Expression and Protein Sequencing, ROS: Reactive oxygen species, scRNA-seq: Single-cell RNA sequencing, TACE: Trans arterial chemoembolization, TAMS: Tumor-associated macrophages, TGF-β: Transforming growth factor-β, Th17: T helper lymphocytes, TME: Tumor microenvironment, Tregs: Regulatory T cells, VEGF: Vascular epithelial growth factor, α-SMA: α-smooth muscle actin.
